# Shorter telomere length in children with autism spectrum disorder is associated with oxidative stress

**DOI:** 10.3389/fpsyt.2023.1209638

**Published:** 2023-06-02

**Authors:** Tong Zhang, Yanan Sun, Jing Wei, Guoqiang Zhao, Wenqi Hao, Zhihai Lv, Xiaohang Chen, Yanan Liu, Fengxiang Wei

**Affiliations:** ^1^Longgang District Maternity and Child Healthcare Hospital, Shenzhen, China; ^2^Department of Maternal, Child and Adolescent Health, School of Public Health, Anhui Medical University, Hefei, China; ^3^The First Affiliated Hospital of Jiamusi University, Jiamusi, China

**Keywords:** Autism spectrum disorder, telomere length, digital PCR, oxidative stress, superoxide dismutase

## Abstract

**Objective:**

Autism spectrum disorder (ASD) is a highly heterogeneous neurodevelopmental disorder caused by a complex interaction between genetic and environmental risk factors. The balance between antioxidant capacity and oxidative stress (OS) induced free radicals may be crucial during the pathophysiological development of ASD.

**Methods:**

In this study, 96 children with ASD who met the diagnostic and statistical manual of mental disorders were collected, and the number of children in the typical development (TD) group was matched by 1:1. Digital PCR (dPCR) for telomere length (TL) expression in ASD in peripheral blood leukocytes. Urine levels of 8-hydroxy-2-deoxyguanosine (8-OHdG) content were measured by tandem triple quadrupole mass spectrometry and corrected by urinary creatinine levels. The levels of superoxide dismutase (SOD), catalase (CAT), and capacity (AOC) were detected by kits.

**Results:**

The TL of the ASD group was shorter than the TD group (*p* < 0.01) and had some accurate predictive significance for the identification of ASD (AUC = 0.632, 95% CI: 0.533–0.710, *p* = 0.002). Both 8-OHdG content and SOD activity in the ASD group were significantly higher than those in the TD group (*p* < 0.05). Shortened TL (Monofactor: 2.20 (1.22, 3.96), *p* = 0.009; Multifactor: 2.22 (1.22, 4.00), *p* = 0.008) and reduced CAT activity (Monofactor: 2.31 (1.28, 4.17), *p* = 0.006; Multifactor: 2.31 (1.28, 4.18), *p* = 0.006) are risk factors for the development of ASD, while reduced 8-OHdG content (Monofactor: 0.29 (0.14, 0.60), *p* = 0.001; Multifactor: 0.27 (0.13, 0.57), *p* = 0.001) and reduced SOD activity (Monofactor: 0.55 (0.31, 0.98), *p* = 0.042; Multifactor: 0.54 (0.30, 0.98), *p* = 0.042) are protective factors for the development of ASD.

**Conclusion:**

In this study, TL and OS were significantly different between the ASD group and the TD group. As guanine-rich telomere sequences were likely damaged by oxygen free radicals, creating OS, which is a factor in the incidence and progression of ASDs. In conclusion, oxidative damage occurs in the bodies of children with ASD, which may lead to sustained disease progression and severe clinical manifestations. We assume that timely supplementation of antioxidants is very likely to be a potential treatment for early intervention in children with ASD. Identification and detection of OS-related biomarkers may contribute to early diagnosis and timely interventions in young patients with ASD.

## 1. Introduction

Autism spectrum disorder (ASD) is a highly heterogeneous neurodevelopmental disorder that occurs in early childhood. The core symptoms are mainly social disorders, language communication disorders, a narrow range of interests or activities, and repetitive behavior (1). In recent years, its prevalence has been increasing (2).

In recent years, studies have found that the increase of intracellular reactive oxygen species (ROS) and reduced free radical damage can easily lead to a reduction in endogenous antioxidant protection system capacity and an oxidative stress (OS) imbalance. This will promote the secretion of vascular and pro-inflammatory molecules and lead to neuroinflammation (3). These oxidative damage processes exist in a wide range of mental diseases. It is reported that OS in the pathological process plays an important role in ASD (4). Currently, it has been discovered that the mechanism of OS hyperactivity is connected to the harm brought on by the interaction between hereditary factors and harmful substances in the internal and external environment. As a result, the pathophysiological development of ASD may depend on how well antioxidant capacity (AOC) and OS-induced free radicals are balanced (5). When OS and mitochondrial dysfunction cause excessive production of ROS, oxidative damage to macromolecules, insufficient energy supply, abnormal mitochondrial signaling function, and other negative effects (6). They destroy the balance of apoptosis, mitochondrial biology, and biogenesis (7). Thus, promoting a variety of clinically related diseases and the aging process (8). Similarly, if the body’s oxidation burden makes it easy to produce a large number of free radicals, nucleic acids, proteins, and lipids are essential cellular components vulnerable to damage. It further causes oxidation of amino acid side chains, cross-linking of protein–protein, oxidation of the protein skeleton, protein fragmentation (9), toxic effects on neurons, a decline in antioxidant levels, and an oxidant ratio imbalance (10).

8-hydroxy-2-deoxyguanosine (8-OHdG) is one of the most common biomarkers of oxidative DNA damage caused by endogenous and exogenous factors (9). Superoxide radicals, an aggressive ROS in aerobic metabolites, can be produced by single-electron reduction of oxygen in the electron transport chain. Superoxide is converted to H_2_O_2_ by SOD, and H_2_O_2_ can be converted to a highly active hydroxyl radical by the Fenton reaction, causing lipid peroxidation (10). The resulting H_2_O_2_ must then be detoxified by CAT’s antioxidant enzymes and broken down into water and oxygen molecules. Therefore, SOD, CAT, 8-OHdG, AOC, and other antioxidants are used as potential biomarkers to evaluate the degree of oxidative damage in the body, which is of great significance to study the association between children with ASD and OS and evaluate the effect of antioxidants on oxidative damage.

Telomere length (TL) is considered a biomarker of cumulative intracellular OS load, which changes the functional status of intracellular telomeres (11). Telomeres are composed of the repetitive non-coding deoxynucleotide sequence TTAGGG, which covers the chromosomes to protect the DNA. TL is influenced by genetic and environmental factors, and TL shortening is strongly associated with cognitive dysfunction due to multiple neuropsychiatric disorders, age, and early life stress (12). In this study, digital PCR (dPCR) for absolute quantification of TL in peripheral blood was used to analyze the correlation between TL and OS biomarkers in children with ASD and to find biomarkers conducive to the early diagnosis of ASD, which is expected to provide guiding suggestions for individualized intervention.

## 2. Method

### 2.1. Study design

Based on the sample size calculation method of cross-sectional studies, the current prevalence rate of ASD children in the world is 1%. If a statistically significant result can be obtained at the level of α = 0.05 and the error is less than 2%. Therefore, at least 95 cases in the study population should be calculated according to the formula below to obtain a statistically significant conclusion.


$$n={\left(\frac{Z_{1}-\frac{\alpha }{2}}{\delta }\right)}^{2}\times p\times \left(1-p\right)$$

In this study, 96 children with ASD who were diagnosed at the Maternal and Child Health Hospital in Longgang District, Shenzhen, from November 2021 to December 2022 were included as an ASD case group, aged 1–10 years. The ethical number is LGFYYXLLL-2021-003. The subject met the diagnostic criteria for ASD in the Diagnostic and Statistical Manual of Mental Disorders (DSM-5). The Autism Behavior Checklist (ABC) and the Childhood Autism Rating Scale (CARS) were used for the clinical evaluation. With respect to sex and age, 96 children in the TD group were matched 1:1 with patients in the ASD group who did not have any neurological conditions, developmental delays, or behavioral traits associated with ASD. The Longgang District Maternal and Child Health Hospital in Shenzhen’s ethical review committee gave their blessing to this study, and each subject’s legal guardian gave their informed consent.

### 2.2. TL Was determined by dPCR

Within 2 h of collection, mononuclear cells were isolated from 1 mL of peripheral blood using a lymphocyte suspension solution (Solaibao, Beijing, China). Cell DNA was extracted using the magnetic bead isolation method and performed on the Lab-Aid 820 Nucleic Acid Extractor (Xiamen Zhishan Biological Technology Co., Ltd., China). The subsequent experimental conditions were met after measuring the sample DNA concentration with the Qubit instrument. In this study, the dPCR instrument (Beijing Xinyi Biological Co., Ltd.) was used to detect TL target genes. The reaction system was 30 μL. The reaction procedure was 95°C 3 min, 95°C 15 s, 55°C 20s, 72°C 25 s, and 40 cycles.

### 2.3. 8-OHdG was detected by LC–MS/MS

Add 100 μL of urine to 800 μL of 0.1% formic acid water and 100 μL of ^15^N_5_-8-OHdG (200 ng/μL) and mix evenly for testing. Internal standard quantification was used, and the 8-OHdG concentration gradient was set to 0.1–50 ng/mL, corresponding to an internal standard concentration of 30 ng/mL. The 8-OHdG content in urine samples was determined by LC–MS/MS (SCIEX QTRAP 6500) and validated using urinary creatinine levels. Phase A is 0.1% formic acid in water, and phase B is a methanol solution. Spiking recovery was used to monitor and verify the feasibility of the sample pretreatment method and the accuracy of the experimental data.

### 2.4. CAT, SOD, and AOC detection

The kit purchased from the Nanjing Institute of Construction Engineering is used to measure the activity of OS markers in the blood. The absorbance values were measured using a microplate reader and UV spectrophotometer, and the corresponding activity was calculated.

### 2.5. Internal verification

We used the ‘caret’ package of R software to conduct two random samplings of 96 patients in the ASD group and 96 children in the TD group. 70% of the patients (n_ASD_ = 68, n_TD_ = 68) were selected as the training set and testing set, respectively. Subsequently, we analyzed the differences in TL and SOD in the ASD and TD groups and the ROC curves in the training and testing sets.

### 2.6. Statistical analysis

SPSS 25.0 statistical software was used for data analysis. Continuous variables were expressed as mean and standard deviation (SD) and compared with Students t-test. Categorical variables are presented as frequency and percentage and were compared with chi-square tests. Risk factors for ASD were analyzed by logistic regression. The receiver operating characteristic (ROC) curve was used to evaluate the diagnostic effect of TL in children with ASD. A partial correlation exists between OS markers level and TL, ABC, and CARS scale scores. All statistical tests were bilateral, and *p* < 0.05 was considered statistically significant.

## 3. Result

### 3.1. Basic data characteristics of the research object

This study included 96 patients in the ASD group and 96 children in the TD group (Table 1). Among them, 81 were boys, with a ratio of 5.4 to 1. There was no significant difference in age (ASD: 4.240 ± 1.840, TD: 4.208 ± 1.045), gender and BMI (ASD: 16.968 ± 4.474, TD: 15.979 ± 3.603) between the two groups (*p* > 0.05), suggesting a reasonable match of subjects. The core symptoms of children in the ASD group were mainly manifested in communication and language impairment, and the proportion of children with mild and moderate abnormalities was larger (Supplementary Tables S1 and S2).

**Table 1:** Basic data on children in the ASD and TD groups (Mean ± SD, *N* (%)).

Sample index	ASD (*N* = 96)	TD (*N* = 96)	t/x^2^	*p*
Age (years)	4.240 ± 1.840	4.208 ± 1.045	0.145	0.885
>4	36 (37.50)	51 (53.125)	4.729	0.030^*^
≤4	60 (62.50)	45 (46.875)		
Gender				
Male	81(84.375)	81(84.375)	0.000	1.000
Female	15(15.625)	15(15.625)		
Height (m)	1.079 ± 0.137	1.088 ± 0.061	0.550	0.583
Weight (kg)	21.000 ± 8.641	18.853 ± 3.897	1.428	0.155
BMI (kg/m^2^)	16.968 ± 4.474	15.979 ± 3.603	1.689	0.093

BMI, Body Mass Index. ^*^p < 0.05.

### 3.2. The difference between two groups of TL and its diagnostic value for ASD

**Figure 1:**
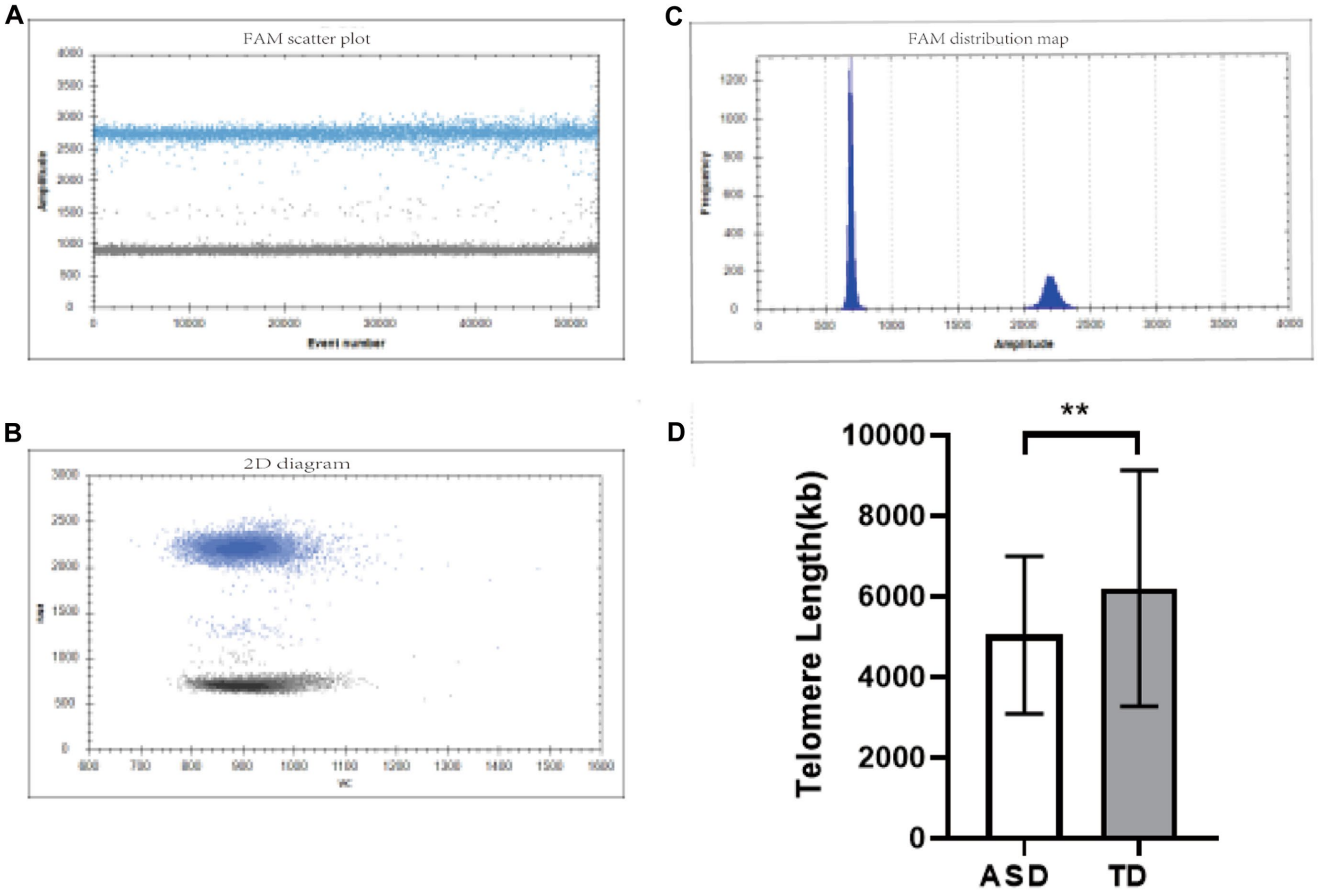
TL detection diagram. **(A,B)** One-dimensional fluorescence scatter plots of the TL gene detected by dPCR. **(C)** Two-dimensional fluorescence scatter plot of the TL gene detected by dPCR. **(D)** Comparison of TL in different groups. ^**^p < 0.01.

The dPCR detection of TL gene fluorescence scatter results showed that the Yin and Yang cell groups were clearly partitioned, and multiple droplet copy numbers could be seen (Figures 1A,B). The absolute quantitative TL in the ASD group was significantly lower than that in the TD group (ASD: 5042.068 ± 1950.595 kb, TD: 6199.267 ± 2931.947 kb, *p* = 0.002) (Figure 1C).

### 3.3. Comparison of OS activity levels between the two groups

**Figure 2:**
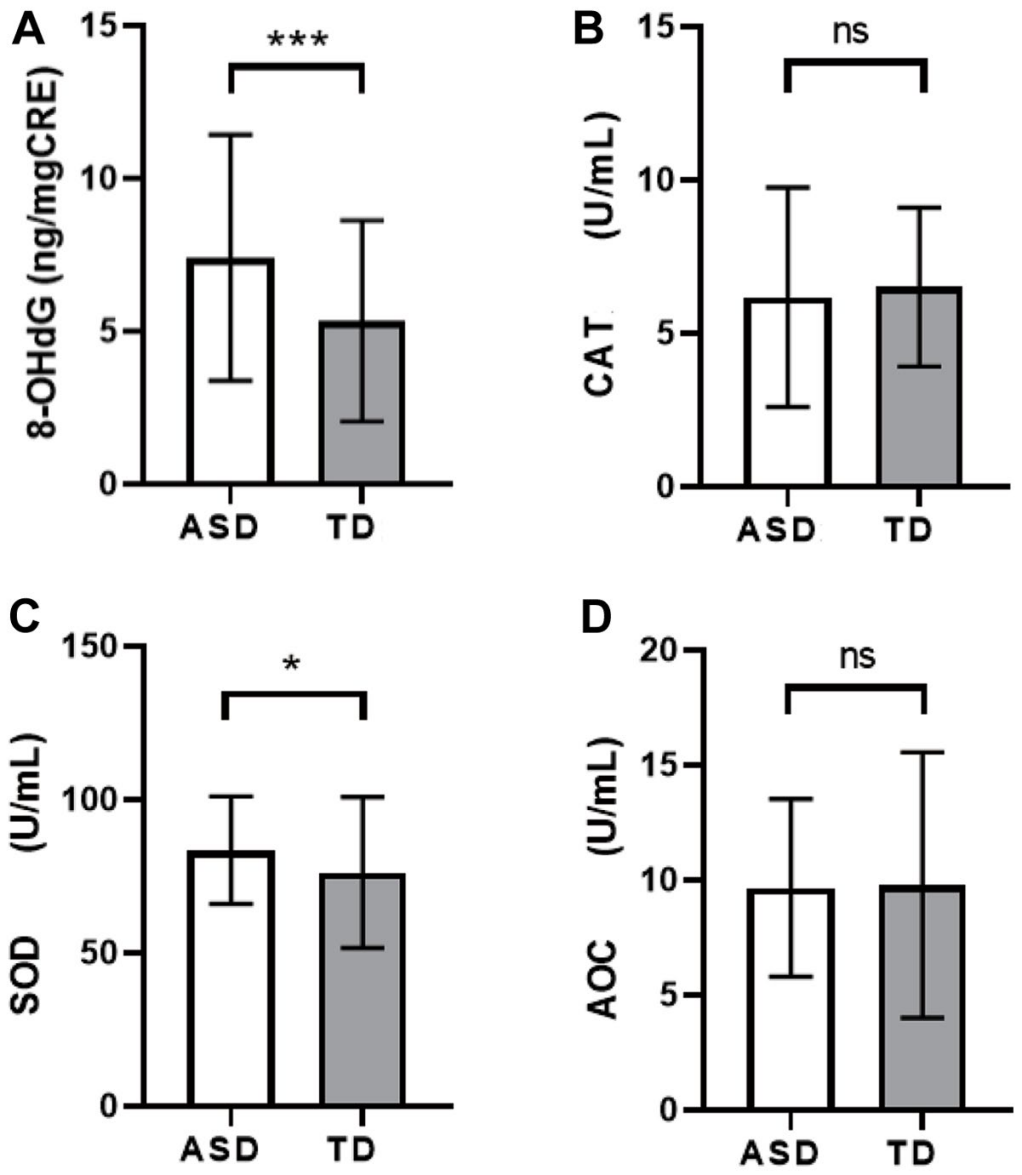
Comparison of levels of OS markers among different groups. **(A)** 8-OHdG. **(B)** CAT. **(C)** SOD. **(D)** AOC. ^*^p < 0.05, ^***^p < 0.001.

The comparison of OS activity levels showed that the 8-OHdG content in the ASD group was higher than that in the TD group (Figure 2A, ASD: 7.41 ± 4.02, TD: 5.37 ± 3.29, *p* < 0.001), and the CAT activity was lower than that in the TD group (Figure 2B, ASD: 6.18 ± 3.58, TD: 6.51 ± 2.59, *p* > 0.05), SOD activity was higher than that of the TD group (Figure 2C, ASD: 83.50 ± 17.57, TD: 76.16 ± 24.60, *p* = 0.019), but there was no significant difference in AOC activity (Figure 2D, ASD: 9.65 ± 3.86, TD: 9.78 ± 5.77, *p* > 0.05).

### 3.4. Internal verification

**Figure 3:**
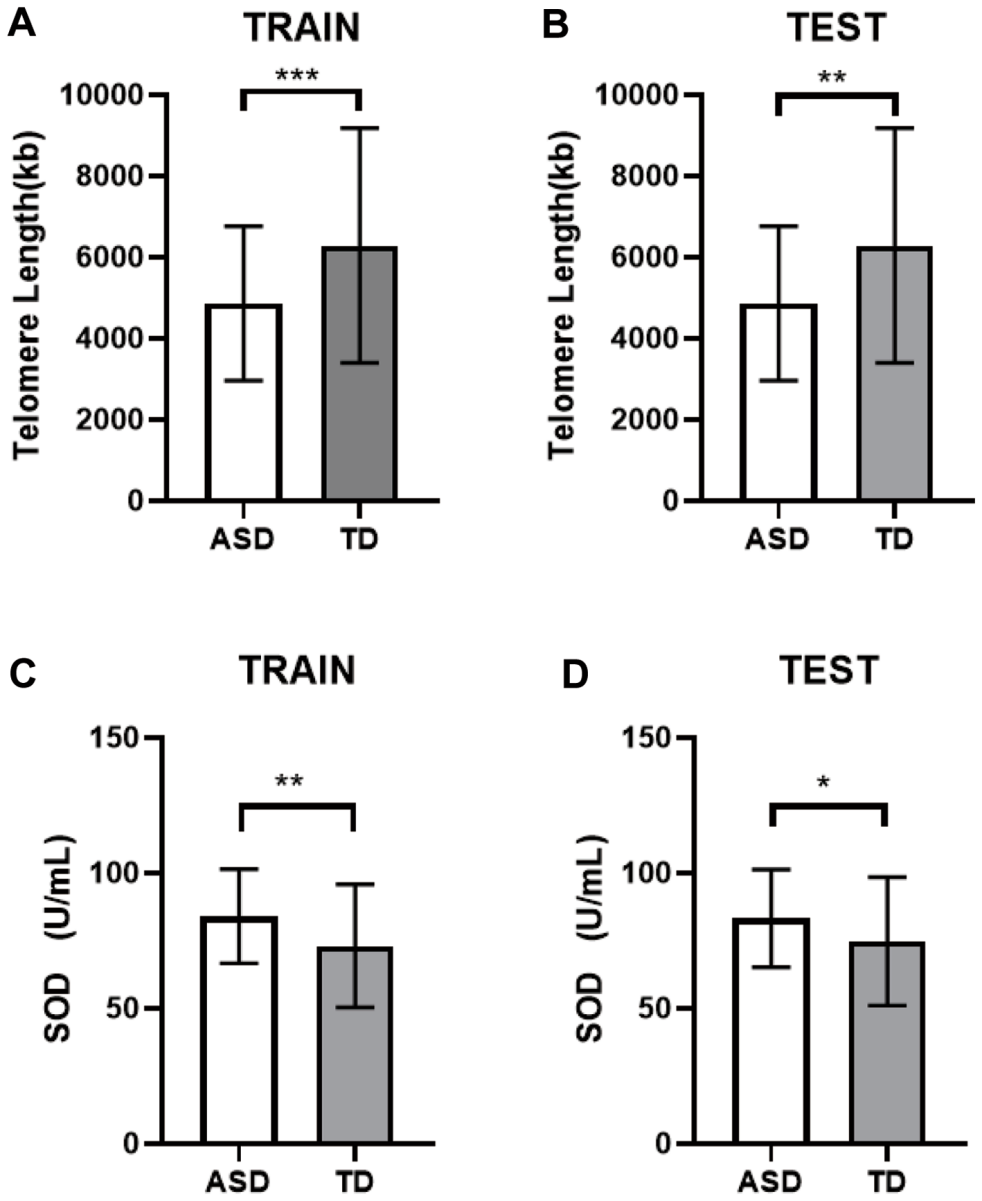
Differential analysis of the internal validation. **(A)** The TL of the training set. **(B)** The TL of the testing set. **(C)** The SOD of the training set. **(D)** The SOD of the testing set. ^*^ *p* < 0.05, ^**^ *p* < 0.01, ^***^ *p* < 0.001.

The TL and SOD contents of the two groups were randomly divided into the training sets and the test sets. Results consistent with the above results showed that the TL in the ASD group was still shorter than that in the TD group (Figures 3A,B), and the SOD activity level was higher than that in the TD group (Figures 3C,D). Both were statistically significant (*p* < 0.05).

**Figure 4:**
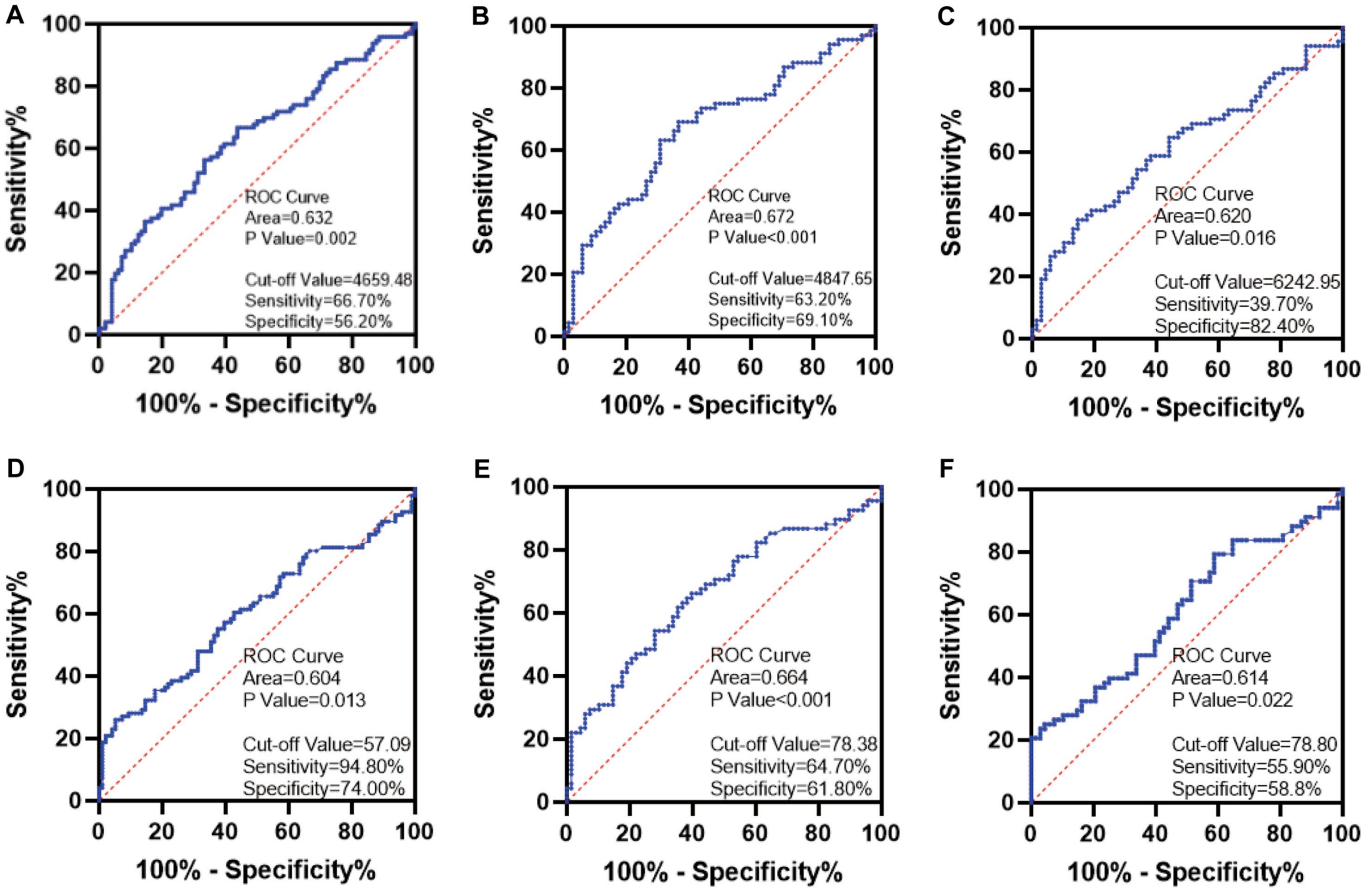
ROC curve plots. **(A)** TL. **(B)** The TL of the training set. **(C)** The TL of the testing set. **(D)** SOD. **(E)** The SOD of the training set. **(F)** The SOD of the testing set.

ROC curve analysis showed the AUC for TL identification of the two groups of children was 0.632 (95% CI: 0.533–0.710), *p* = 0.002. By calculating the cut-off value, the optimal cut-off value was 4659.48 kb, and the sensitivity and specificity were 66.70 and 56.20%, respectively (Figure 4A). The results of training set and test set showed that TL had a certain accuracy in diagnosing ASD (Figure 4B: AUC = 0.672 (95% CI: 0.581–0.763), *p* < 0.001; Figure 4C: AUC = 0.620 (95% CI: 0.525–0.715), *p* = 0.016).

The AUC for SOD identification of the two groups of children was 0.604 (95% CI: 0.524–0.685), *p* = 0.013. By calculating the cut-off value, the optimal cut-off value was 57.09 U/mL, and the sensitivity and specificity were 94.80 and 74.00%, respectively (Figure 4D). The results of the training set and testing set showed that SOD activity may be considered as a biomarker for the diagnosis of ASD (Figure 4E: AUC = 0.664 (95% CI: 0.573–0.756), *p* < 0.001; Figure 4F: AUC = 0.614 (95% CI: 0.519–0.709), *p* = 0.022).

### 3.5. Logisitic regression analysis of the correlation between OS markers and ASD occurrence

The median values of all detection indicators in the TD group were taken as truncation values. The study population was first divided into a long TL group (> 5481.30) and a short TL group (< 5481.30) and assigned a value of 0 or 1. The results showed the number and percentage of cases in the two groups grouped by truncation value (Table 2). The association between TL and risk of ASD was analyzed by univariate logistic regression, and the OR and 95% CI of TL detected by dPCR were 2.20 (1.22, 3.96), *p* = 0.009, suggesting that patients in the short TL group were at greater risk of ASD. After adjusting for gender and age, the OR and 95% CI of multiple logistic regression were 2.22 (1.22, 4.00), *p* = 0.008, indicating that the correlation between TL shortening and the occurrence of ASD was statistically significant.

**Table 2:** Monofactor and multifactor logistic regression analysis of OS markers level.

	ASD(%)	TD(%)	Monofactor		Multifactor	
			OR (95%CI)	*p* value	OR (95%CI)	*p* value
TL						
≥5481.30	30 (31.25)	48 (50.00)	1.00 (reference)		1.00 (reference)	
<5481.30	66 (68.75)	48 (50.00)	2.20 (1.22, 3.96)	0.009^**^	2.22 (1.22, 4.00)	0.008^**^
8-OHdG						
>4.63	47 (77.05)	44 (50.57)	1.00(reference)		1.00 (reference)	
<4.63	14 (22.95)	43 (49.43)	0.29 (0.14, 0.60)	0.001^**^	0.27 (0.13, 0.57)	0.001^**^
CAT						
>6.60	29 (30.21)	48 (50.00)	1.00 (reference)		1.00 (reference)	
<6.60	67 (69.79)	48 (50.00)	2.31 (1.28, 4.17)	0.006^**^	2.31 (1.28, 4.18)	0.006^**^
SOD						
>78.12	47 (48.96)	48 (50.00)	1.00 (reference)		1.00 (reference)	
<78.12	49 (51.04)	48 (50.00)	0.55 (0.31, 0.98)	0.042^*^	0.54 (0.30, 0.98)	0.042^*^
AOC						
>9.31	45 (46.88)	48 (50.00)	1.00 (reference)		1.00 (reference)	
<9.31	51 (53.12)	48 (50.00)	1.13 (0.64, 2.00)	0.665	1.14 (0.65, 2.02)	0.649

OR, Odds Ratio; CI, Confidence Interval. ^*^p < 0.05, ^**^p < 0.01.

The truncated value of the 8-OHdG content was 4.63 and assigned: < 4.63 = 0, > 4.63 = 1. Univariate and multivariate logistic regression analysis showed low 8-OHdG levels (univariate: 0.29 (0.14, 0.60), *p* = 0.001; multiple factors: 0.27 (0.13, 0.57), *p* = 0.001); and low activity SOD levels (univariate: 0.55 (0.31, 0.98), *p* = 0.042; multiple factors: 0.54 (0.30, 0.98), *p* = 0.042) were protective factors for ASD, and a low CAT level was a significant risk factor for ASD (univariate: 2.31 (1.28, 4.17), *p* = 0.006; multiple factors: 2.31 (1.28, 4.18), *p* = 0.006). However, a low AOC activity level may be a risk factor (*p* > 0.05), and more sensitive detection methods should be used to identify the association between the two groups.

### 3.6. Correlation of OS activity level, TL and scores of ABC and CARS

**Figure 5:**
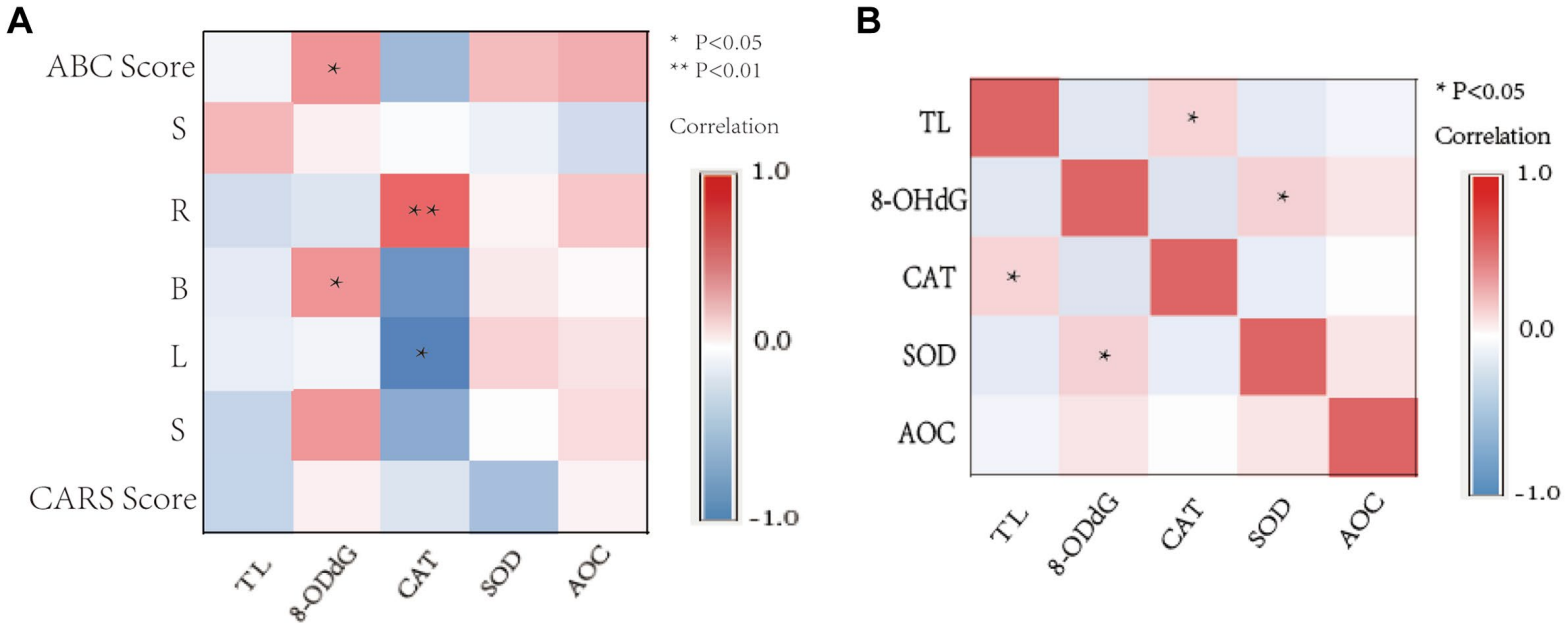
Correlation heat map. **(A)** Correlation between OS activity levels, TL, ABC, and CARS scale scores in children with ASD. **(B)** Correlation between TL and OS activity levels in children with ASD. S, Sensory; R, Relating; B, Body and object use; L, Language; S, Social and self-help. ^*^p < 0.05, ^**^ *p* < 0.01.

Partial correlation analysis (adjusting for age and gender variables) was used to evaluate the relationship between TL, 8-OHdG, CAT, SOD, AOC, and ABC and CARS scores in the ASD group (Figure 5A). The 8-OHdG level of the OS index was significantly correlated with the total score of the ABC scale (*r* = 0.230, *p* = 0.029), physical exercise ability (*r* = 0.232, *p* = 0.027), and self-care ability (*r* = 0.227, *p* = 0.032). CAT activity level was significantly correlated with communication ability (*r* = 0.297, *p* = 0.004) and positively correlated with language ability (*r* = −0.240, *p* = 0.023). There was no significant correlation between the level of other OS indexes and the total scores of ABC, CARS, and all scores (*p* > 0.05).

### 3.7. Correlation between the level of OS markers and TL

The Spearman correlation results showed that TL was positively correlated with CAT activity (*r* = 0.246, *p* = 0.016) (Figure 5B), and 8-OHdG content was positively correlated with SOD activity (*r* = 0.262, *p* = 0.012). There was no significant correlation among other OS markers (*p* > 0.05).

## 4. Discussion

The subjects included in this study are infants and children, and the age structure is relatively uniform. Among ASD children, the ratio of male to female is about 5:1, which is consistent with the conclusion of the autism network monitoring report that it is more likely to occur in males (13). The core symptoms of children with ASD are mainly weak social communication ability and language retardation, followed by symptoms of sensation, physical movement, and self-care. Most children have mild to moderate abnormalities, which is consistent with the conclusion of Tereshko et al. (14). This study discussed the expression and clinical significance of TL and OS markers in ASD children, and it analyzed the correlation between TL and disease severity.

The results showed that the TL in peripheral blood leukocytes in the ASD group was shorter compared with that of the TD group. Telomeres are repetitive sequences of non-coding deoxynucleotides that cover chromosomes to protect DNA. TL is influenced by the interaction of both genetic and environmental factors, and TL shortening is associated with a variety of neuropsychiatric diseases, early-life stress, and age-related cognitive dysfunction (15). Results showed that TL shortening was a risk factor for ASD, which was consistent with Li et al. (16).The underlying biological mechanism of telomere shortening may be associated with excessive immune cell mitosis (possibly repeated clonal expansion in leukocytes), increased OS and inflammatory response, reduced brain growth factors, metabolic factor imbalance, and dysfunction of the hypothalamic–pituitary–adrenal axis and autonomic nervous system (17). Logistic regression analysis showed that TL shortening was a risk factor for the occurrence of ASD, suggesting that TL might be closely associated with developmental delay and symptom aggravation in children with ASD, which was consistent with Lewis et al.’s finding that TL shortening was associated with more severe sensory symptoms (15).

In this study, dPCR was used to quantify the DNA of peripheral blood cells. This new PCR detection technology provides a simple workflow, and the method of separating different DNA templates can improve amplification efficiency and achieve simultaneous amplification of different samples so that the target nucleic acid in the sample can be accurately quantified (18). The dPCR technology does not require a standard dilution curve, reaction efficiency, or reference samples and can directly detect the copy number of target sequences. It is a highly accurate and precise detection method (19), but its cost of instruments, reagents, and consumables is higher than that of qPCR technology.

The results of logistic regression analysis showed that decreased CAT activity was a risk factor for the occurrence of ASD and decreased 8-OHdG content and SOD activity were protective factors for the occurrence of ASD. There was a synergistic effect between the two, indicating that the body may be in the process of oxidative damage and oxidative damage could easily lead to the sustainable development of the disease and serious clinical manifestations. This result is consistent with the study of Hao Zhou et al., which indicated that the balance between antioxidant capacity and OS-induced free radicals is the key to the pathophysiological development of ASD (5). In this study, only four representative indicators were selected, which could not represent the overall oxidation capacity level of the body.

8-OHdG is produced by the oxidation reaction of a large number of ROS attacking the 8th carbon atom of the DNA guanine base and can exist stably *in vivo*, which is considered the most commonly used biomarker for oxidative damage to DNA (20). However, due to the young age of the subjects included in this study and their poor control of excretory organs, some children could not collect urine samples for retention and detection of 8-OHdG content. But in this study, LC–MS/MS detection with high selectivity and specificity was adopted, which, compared with previous ELISA techniques (21), made up for the deficiency of specificity caused by cross-reaction between antibodies and reflected the content of 8-OHdG in the body more accurately and quickly. The results of this study showed that the 8-OHdG level of children in the ASD group was significantly higher than that in the TD group. Therefore, the identification and detection of OS-related biomarkers such as abnormal increases in active substances and antioxidant defense against free radicals may contribute to early diagnosis and timely interventions in young patients with ASD.

The results of this study showed that the SOD activity of the ASD group was higher than that of the TD group, and the CAT activity level was similar to that of the TD group. This was consistent with the results of Al-Gadani et al., who found that SOD overexpression was observed in ASD patients and CAT remained unchanged (22). However, Yenkoyan et al. found that SOD/CAT imbalances occurred in peripheral blood polymorphonuclear leukocytes (23). This may be due to continuous inflammation, which eventually leads to abnormal function of neuron cells or neuron death. The AOC activity level in the ASD group was slightly decreased, but no significant difference was found compared with that in the TD group, which should be further increased in sample size or with more accurate research methods for detection.

The internal verification results of the training set and test set were consistent with the differences in results of TL and SOD activity between the two groups and had significant statistical significance. ROC curve analysis suggested that TL activity might be considered a biomarker reflecting the early diagnosis and prediction of ASD. At the same time, SOD activity has a certain accuracy in the diagnostic value of ASD. In this study, the number of participants in 8-OHdG detection was small, so in order to avoid overgeneralization, we did not analyze the ROC curve of 8-OHdG. Overall, the regulation and detection of potential biomarkers for OS is an important aspect of pathophysiology and mitochondrial genetics, contributing to targeted disease prevention, early diagnosis, and individualized therapy.

Partial correlation analysis showed that 8-OHdG content and CAT activity were closely related to the severity of ASD. This confirms that there is a significant imbalance of oxygen supply and demand in ASD, and the detection of OS-related biomarkers is of great significance for the assessment of oxidative damage in the body. It also suggests that antioxidant supplementation may be a potential treatment for early intervention in children with ASD. Spearman’s correlation analysis showed that TL was positively correlated with CAT activity. This indicates that guanine rich in telomere sequence is highly likely to have been attacked by oxygen free radicals to produce OS, and due to the lack of a repair mechanism after telomere sequence damage, TL will shorten continuously with the increase in the number of divisions and thus participate in the occurrence and development of ASD. However, no relationship between TL and 8-OHdG was found in this study. On the one hand, it may be due to the small amount of 8-OHdG adduct combined with guanine and hydroxyl radicals in the occurrence of OS in the body, resulting in a few double-strand breaks of telomere sequences; on the other hand, it may be due to the small number of subjects included in this study, so it is necessary to further increase the sample size to confirm this hypothesis.

In this study, no significant differences were found in some indicators, so it is possible to increase the sample size or adopt more sensitive and accurate detection methods to further confirm the theory of oxygen supply and demand imbalance in the body. In the future, it is expected to analyze the risk factors and pathogenesis of ASD through cell experiments, prospective cohorts, and other further studies and further explore the etiology and pathophysiological processes of ASD so to provide application value for early intervention and scientific treatment.

Overall, this study adopted a more sensitive and accurate dPCR detection method for absolute quantification of samples with low copies of target molecules and small template concentration differences. There were significant differences in TL and SOD activity between the two groups of children, which had certain diagnostic value for ASD. The decrease in 8-OHdG content, the increase in CAT activity, and the decrease in SOD activity indicate that the body is in the process of oxidative damage, which is a risk factor for the occurrence of ASD and is closely related to the severity of the disease. Telomere sequence deletion and structural destruction in the body were significantly correlated with increased CAT activity. This suggests that supplementation of antioxidants to remove free radicals to protect cells and thus reduce oxidative damage may be a new idea to assist clinical diagnosis and treatment. This provides a theoretical basis for the occurrence, development, and pathogenesis of ASD.

## Data availability statement

The original contributions presented in the study are included in the article/Supplementary material, further inquiries can be directed to the corresponding author.

## Ethics statement

This study has been reviewed and approved by the ethical review committee of the Longgang District Maternal and Child Health Hospital in Shenzhen (ethical number: LGFYYXLLL-2021-003). The legal guardians of all subjects signed informed consent to participate.

## Author contributions

TZ conducted the literature search and drafted the manuscript. YS and YL wrote part of the manuscript. ZL conducted pre-enrollment advocacy for children with ASD. JW assessed the scale on the children. XC and WH offer new ideas for problem solving and grammar modification. GZ and FW supervised the entire process, and revised the manuscript. All authors contributed to the article and approved the submitted version.

## Funding

Basic research project of Shenzhen Science and Technology Research Projects (JCYJ20220530162412029) and Shenzhen City and Longgang District Science and Technology Innovation Projects (LGKCYLW2022008).

## Conflict of interest

The authors declare that the research was conducted in the absence of any commercial or financial relationships that could be construed as a potential conflict of interest.

## Publisher’s note

All claims expressed in this article are solely those of the authors and do not necessarily represent those of their affiliated organizations, or those of the publisher, the editors and the reviewers. Any product that may be evaluated in this article, or claim that may be made by its manufacturer, is not guaranteed or endorsed by the publisher.
