# Supplementary material for: Shorter telomere length in children with autism spectrum disorder is associated with oxidative stress
Source: Front Psychiatry. 2023 Jun 2;14:1209638. doi: 10.3389/fpsyt.2023.1209638 (PMC10272824; doi:10.3389/fpsyt.2023.1209638)
Supplement: Supplementary file 1 [file Table_1.DOC]

# Table S1 The ABC scale scores of the ASD group.

| ABC Score | ASD Group(N=96，Mean ± SD) |
| --- | --- |
| S | 7.896±4.366 |
| R | 16.458±5.966 |
| B | 6.521±6.283 |
| L | 13.917±5.943 |
| S | 9.792±4.570 |
| Score | 54.365±14.924 |

S: Sensory; R: Relating, B: Body and object use, L: Language, S: Social and self-help.
