# Supplementary material for: Shorter telomere length in children with autism spectrum disorder is associated with oxidative stress
Source: Front Psychiatry. 2023 Jun 2;14:1209638. doi: 10.3389/fpsyt.2023.1209638 (PMC10272824; doi:10.3389/fpsyt.2023.1209638)
Supplement: Supplementary file 2 [file Table_2.DOC]

Table S2 The CARS scale scores of the ASD group.

| CARS (Score) | ASD Group (N=96，N(%)) | ASD Group (Mean ± SD) |
| --- | --- | --- |
| >25.5 | 18（18.750） | 27.056±5.145 |
| 30-36 | 44（45.833） | 31.682±5.072 |
| >36 | 34（35.417） | 39.529±5.174 |
