# Supplementary figures and images for: Shorter telomere length in children with autism spectrum disorder is associated with oxidative stress
Source: Front Psychiatry. 2023 Jun 2;14:1209638. doi: 10.3389/fpsyt.2023.1209638 (PMC10272824; doi:10.3389/fpsyt.2023.1209638)

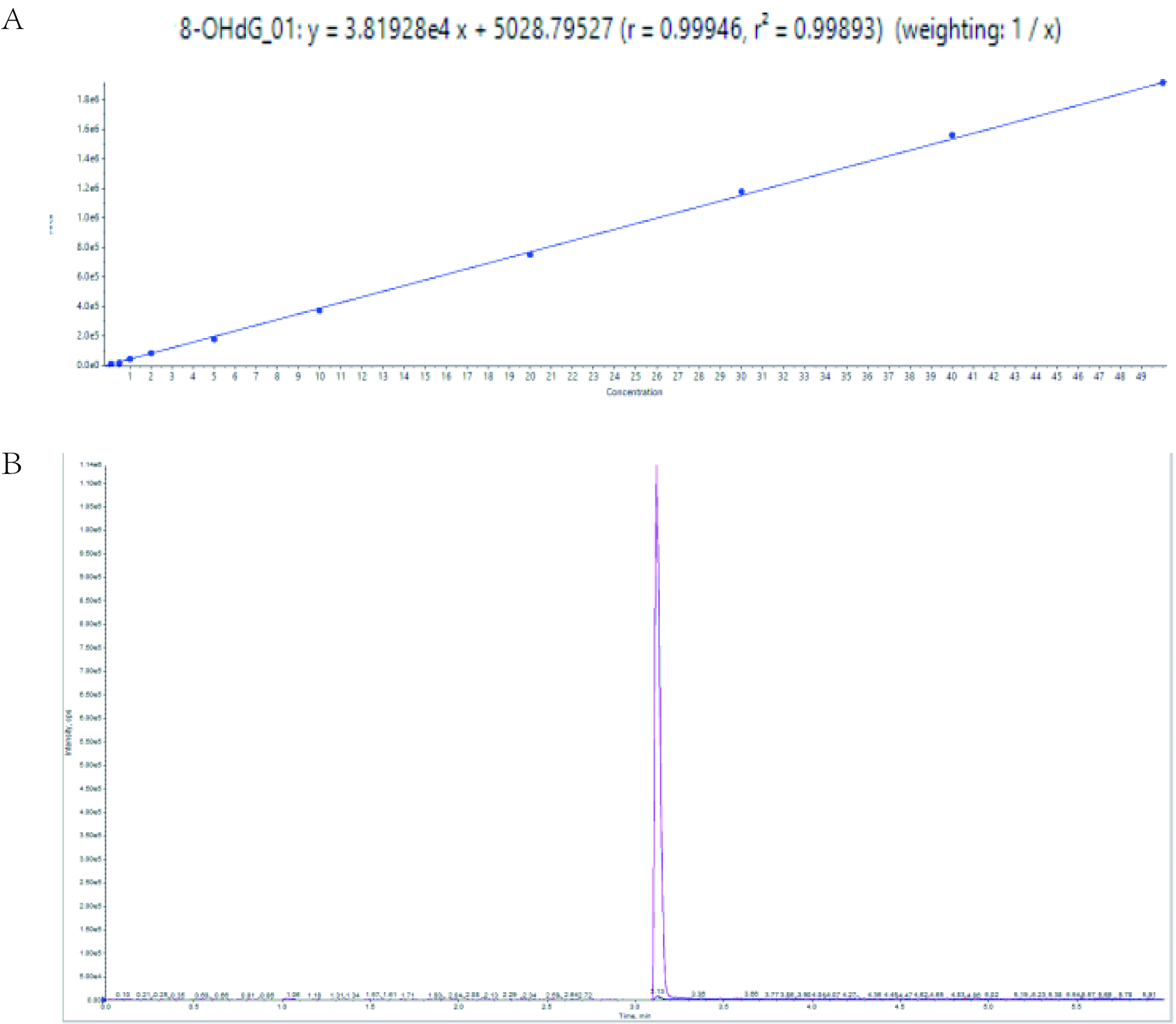

Supplement: Supplementary file 3 [file Image_1.TIF]
